# Effectiveness of a smoking cessation educational program for Japanese nurses on subsequent changes of behavior in delivering smoking cessation counseling

**DOI:** 10.18332/tid/144649

**Published:** 2022-02-18

**Authors:** Chie Taniguchi, Izumi Sezai, Itsuro Yoshimi, Tomoyasu Hirano, Fumihiko Wakao

**Affiliations:** 1 College of Nursing, Aichi Medical University, Nagakute, Japan; 2 National Cancer Center, Tokyo, Japan; 3 Community Health Nursing Section, National Defense Medical College, Tokorozawa, Japan

**Keywords:** nurses, smoking cessation, education program, behavioral change

## Abstract

**INTRODUCTION:**

Despite the effectiveness of smoking cessation counseling, participation of nurses in delivering smoking cessation advice has been far from satisfactory in practice. Training nurses is considered to be important for increasing self-efficacy and skills for routine delivery of smoking cessation counseling. The aim of the present study was to evaluate the effectiveness of a smoking cessation educational program for Japanese nurses on subsequent changes of their behavior in delivering smoking cessation counseling, three months later.

**METHODS:**

We ran a 6-hour smoking cessation educational program for nurses recruited from the Nursing Associations of 13 prefectures in Japan between May 2019 and February 2020. Surveys were conducted by questionnaire before the start of the program and 3 months thereafter. The successful implementation of smoking cessation counseling behavior was evaluated according to the 5As of the Clinical Practice Guidelines for Treating Tobacco Use and Dependence (Ask, Advise, Assess, Assist, Arrange).

**RESULTS:**

We received 289 responses 3 months after the program finished (response rate 46.0%). At that time, 43% of participants had increased the frequency of ‘Ask’ and 42.1%, 50%, 39.3%, and 28.6%, respectively, had also increased their frequency of ‘Advise’, ‘Assess’, ‘Assist’, and ‘Arrange’. We found that smoking cessation counseling was significantly more frequently delivered after the educational program for those participants who had routinely delivered ‘Advise’ before the program as measured by increased delivery of ‘Assess’ and ‘Assist’ afterwards (OR=2.39; 95% CI: 1.00–5.69, OR=2.54; 95% CI: 1.16–5.60 and OR=3.68; 95% CI: 1.40–9.65, OR=2.77; 95% CI: 1.10–7.01, respectively).

**CONCLUSIONS:**

The program successfully increased the frequency of nurses providing smoking cessation advice to patients. Readiness to deliver smoking cessation counseling before the program and continuing self-efficacy after the program are important for changing the behavior of nurses in delivering smoking cessation counseling.

## INTRODUCTION

Smoking has a well-known and very substantial impact on death from non-communicable chronic diseases^1^. Smoking cessation significantly reduces the risk of dying from tobacco-related diseases including cardiovascular disease^2^, chronic obstructive pulmonary disease^3^ and cancers^4,5^. It is thus clearly desirable to reduce the prevalence of smoking through successful smoking cessation programs.

Effectiveness of smoking cessation counseling given by nurses has been reported for various sections of the population, such as hospital inpatients, outpatients and those undergoing routine health check-ups^6-9^. A previous meta-analysis that investigated the efficacy of nurses’ intervention for encouraging smoking cessation indicated that it is beneficial for helping patients to quit (OR=1.43; 95% CI: 1.24–1.66)^8^. Despite this documented effectiveness of smoking cessation counseling, many studies suggest that participation of nurses in delivering such advice has been far from satisfactory^10-12^.

Skill training and motivational programs for nurses to encourage the delivery of smoking cessation counseling is likely to be important for increasing the success of this important activity, and for nurses to routinely deliver such advice and to act on it themselves. Previously, we reported the real-world situation of Japanese nurses’ smoking cessation practices^13^. Results suggested that patients treated at institutions in which nurses received smoking cessation counseling skill training showed significantly higher quitting rates than patients nursed by staff who had not received such training^13^. A few other studies have investigated the effectiveness of smoking cessation training for nurses for their subsequent counseling behaviors^14,15^. Borrelli et al.^16^ conducted a one-day smoking cessation training program trial for home healthcare nurses and investigated their attitudes and actual practices 6 months thereafter. They reported that brief training was associated with changes in both attitudes and behaviors regarding smoking cessation counseling^16^. Despite the advantages of long-term follow-up, this study was limited in that it involved only 63 nurses and the training program was not standardized. For these reasons, we decided to perform a trial of a one-day smoking cessation educational program for Japanese nurses and to investigate the effectiveness of the program in influencing the participants’ subsequent behavioral changes in the delivery of smoking cessation advice in practice.

## METHODS

### Study design

This was a longitudinal study using survey questionnaire responses before implementing the smoking cessation educational program and 3 months thereafter.

### Settings

We organized a smoking cessation educational program for nurses between May 2019 and February 2020 with the cooperation of the Nursing Associations in 13 prefectures in Japan. The program consisted of three sessions spaced over six hours. Because this program aimed to assess the delivery of smoking cessation counseling in practice, most of the participants had already provided some smoking cessation support to their patients in the past. Therefore, we focused on the stage of ‘Assess’ and ‘Assist’ in the so-called 5As of the Clinical Practice Guidelines for Treating Tobacco Use and Dependence^17^ as follows:

- First session (1 hour): a physician provided information on the harm of active smoking, passive smoking, merits of quitting smoking, mechanism of nicotine dependence, and knowledge of heated tobacco products (HTPs).
- Second session (2.5 hours): a lecture by a nurse specializing in smoking cessation counseling and brief active learning exercise on providing smoking cessation counseling focusing on ‘Assess’ and ‘Assist’^18^. This session consisted of items dealing with ‘Effectiveness of nurses' counseling for smoking cessation’, ‘Stage of change’, ‘Assessment of stage’, ‘How to increase patients' motivation and self-efficacy of quitting smoking’ and ‘How to make personal quit plan for preparation stage patients’.
- Third session (2.5 hours): workshop on how to advise patients, including skill training by a specialized public health nurse together with a member of the Motivational Interviewing Network of Trainers^19^. This session consisted of items dealing with ‘Motivational interviewing’, ‘Partnership with patients’, ‘Acceptance and compassion’, ‘Strengthen personal motivation’, and ‘Role-play with participants: Smoking cessation counseling for precontemplation stage and contemplation stage patients’.

We arranged a self-administered questionnaire survey just before the nurses participated in the program and 3 months thereafter. The before and after questionnaires were linked by assigning them an identical random number.

### Participants

**Figure 1:**
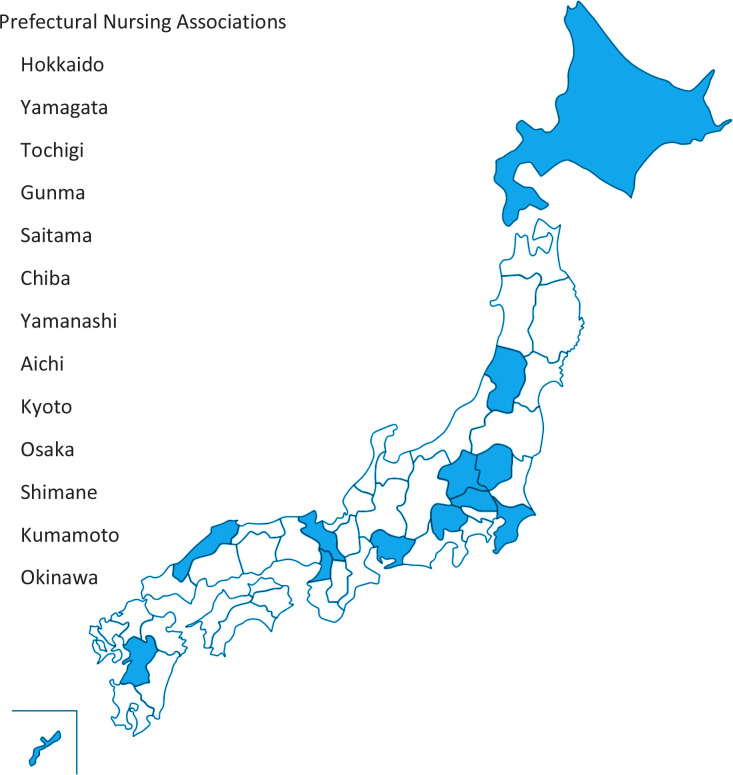
Map showing the 13 prefectural Nursing Associations that participated in the program

We advertised at the 47 prefectural Nursing Associations (NA) for nurses willing to participate in a smoking cessation educational program. Of these 47 associations, the following 13 agreed to cooperate: Yamagata (number of participants, 51), Kumamoto (71), Tochigi (25), Chiba (27), Gunma (25), Hokkaido (71), Yamanashi (50), Osaka (82), Kyoto (37), Okinawa (67), Shimane (35), Saitama (119), and Aichi (22) (Figure 1). Each NA recruited participants for the program by internet or mail. The fee for participation was set by each NA, ranging from about 2000 to 5000 yen. The definition of ‘nurses’ used in this study encompassed those who were nationally qualified or locally qualified in the prefecture, including associate nurses, registered nurses, public health nurses, and midwives.

### Measures and variables

Demographic information included age, sex, type of nurse, educational and nursing histories, information on the workplace, and smoking status. Self-efficacy for smoking cessation counseling was defined as confidence in the ability to counsel patients who smoke. It was rated on a scale of 1 (not at all confident) to 10 (very confident)^16^. Participants whose self-efficacy had increased by at least one point 3 months after the program were defined as the ‘positive group’, and those with no change or a decrease were defined as the ‘negative group’. Smoking cessation practices were defined based on the 5As (Ask, Advise, Assess, Assist, Arrange)^17^. Participants were asked about their frequency of delivering smoking cessation counseling based on the 5As using the five responses ‘always’, ‘usually’, ‘sometimes’, ‘rarely’, and ‘never’. We assigned those who answered ‘always’, ‘usually’ or ‘sometimes’ to an initial ‘positive behavior’ group and those who answered ‘rarely’ or ‘never’ to a ‘negative behavior’ group.

### Statistical analysis

We calculated the frequency of ‘positive behavior’ for each of the 5As 3 months after the program for those participants who were assigned to the ‘negative behavior’ group before the program. To assess factors associated with increased delivery of ‘Assess’ and ‘Assist’ 3 months after the program among these participants, we performed multi-variable logistic regression analysis with adjustment for age, education level (less than vs more than 4 years of nursing education), workplace (health or prevention workplace such as public health center or health check-up center vs medical workplace such as hospital or clinic), changing self-efficacy for smoking cessation counseling before and 3 months after the program (negative vs positive-efficacy group), and implementation status of ‘Ask’, ‘Advise’, ‘Assess’ before the program (negative vs positive). Significance was set at p<0.05. STATA version 16 (STATA Corp, College Station, TX) was used for the analysis.

## RESULTS

### Characteristics of the study subjects

A total of 628 nurses participated in the program, of whom 289 responded to the questionnaire 3 months thereafter (response rate 46.0%). The mean age of the respondents was 45.6±9.8 years ( range: 23–80) (Table 1). Almost 80% of respondents were registered nurses with a national qualification. Over 70% of the respondents worked in medical institutions such as hospitals or clinics. Only 16 respondents (5.6%) were current smokers when they participated in the program (Table 1).

**Table 1:** Characteristics of respondents who participated in a cross-sectional survey, 2019– 2020 (N=289)

*Characteristics*	*n*	*%*	*Mean (SD)*
**Sex**			
Male	13	4.5	
Female	276	95.5	
**Age** (years)			45.6 (9.8)
**Types of nurses**			
Practical	12	4.2	
Registered	223	78.2	
Public health	46	16.1	
Midwife	4	1.4	
**Education level**			
High school	7	2.4	
Vocational school	206	71.3	
Junior college	22	7.6	
College	45	15.6	
Graduate school	4	1.4	
Other	5	1.7	
**Nursing experience** (years)			21.1 (9.8)
**Workplace**			
Hospital	195	67.4	
Clinic	29	10.0	
Public health center	12	4.2	
Health check-up center	25	8.7	
Other	28	9.7	
**Smoking status**			
Current smoker	16	5.6	
Ex-smoker	76	26.4	
Never smoker	196	68.0	
**Self-efficacy for smoking cessation counseling before the program**			3.6 (2.2)

### Delivery of smoking cessation counseling based on the 5As before and 3 months after the program

**Figure 2:**
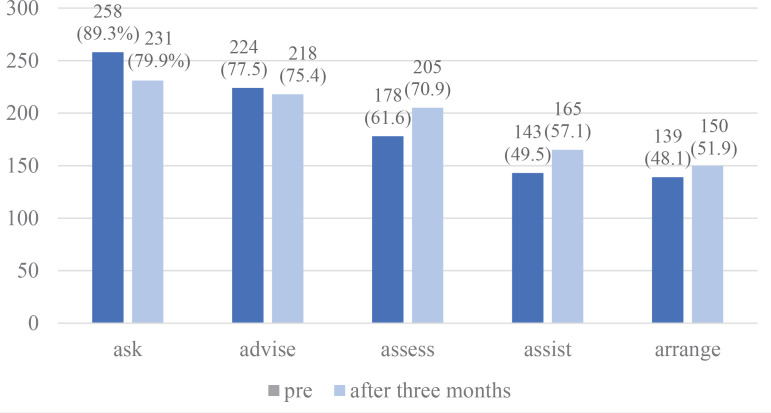
Numbers of participants indicating their delivery of the 5As before and after the educational program

The proportion of participants who had positive behavior for counseling according to the 5As as defined in the Methods is shown in Figure 2. The proportion positive for ‘Assess’ and ‘Assist’ was significantly increased after the program (62% to 71% and 50% to 57%, respectively, p<0.05 for both), whereas ‘Ask’, ‘Advise’ and ‘Arrange’ were not. The proportion of those for whom delivery of the 5As gradually decreased with advancing steps for cessation counseling from ‘Ask’ through to ‘Arrange’ is shown in Figure 2.

### Behavioral change in delivering cessation counseling after participating in the program

**Figure 3:**
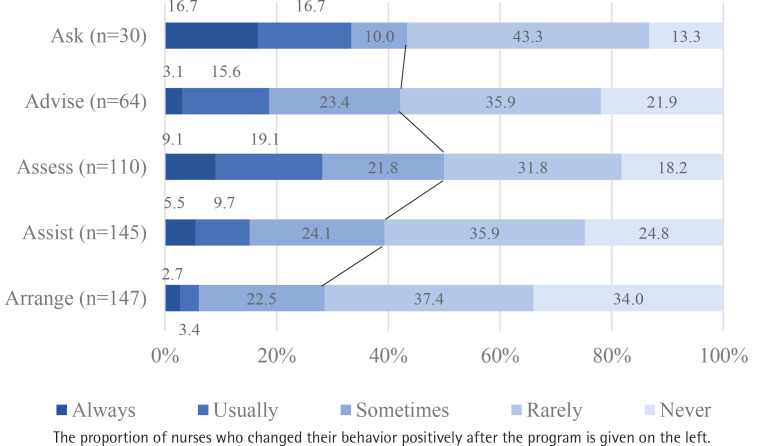
Frequency of smoking cessation counseling at 3 months after the program for those who did not offer smoking cessation counseling before participating in the educational program

Of the participants who had initially given a negative response to the ‘Ask’ question, 43% were positive for this item at 3 months thereafter (Figure 3). At this time, 42.1%, 50%, 39.3%, and 28.6% of nurses who had been negative for ‘Advise’, ‘Assess’, ‘Assist’, and ‘Arrange’ before participating in the program, became positive for this behavior (Figure 3).

### Factors associated with positive post-program changes of smoking cessation counseling practices

We performed logistic regression analysis to identify factors associated with a positive behavioral change for the ‘Assess’ and ‘Assist’ items of the clinical practice guidelines for treating tobacco use and dependence, after adjusting for confounding factors in participants who had infrequently practiced smoking cessation counseling before participating in the program (Table 2). Positive changes of self-efficacy for smoking cessation counseling 3 months after participating in the program were apparent (adjusted odds ratio, AOR=2.39; 95% CI: 1.00–5.69). The delivery of the ‘Advise’ item before the program (AOR=3.68; 95% CI: 1.40–9.65) was associated with increased frequencies of delivery of ‘Assess’. Age (AOR=0.39; 95% CI: 0.17–0.89), self-efficacy (AOR=2.54; 95% CI: 1.16–5.60), and delivery of ‘Advise’ before the program (AOR=2.77; 95% CI: 1.10–7.01) were associated with an increased frequency of delivery of ‘Assist’ thereafter. Also, to compare changing self-efficacy after the program, we performed univariate logistic regression analysis. Positive change after the program was observed for ‘Advise’ and ‘Assist’ (AOR=1.18; 95% CI: 0.42–3.31 and AOR=0.81; 95% CI: 0.33–2.36, respectively).

**Table 2:** Factors associated with post-program change of smoking cessation counseling delivering ‘Assess’ and ‘Assist’ participants (N=289)

		Assess			Assist		
		AOR	p	95% CI	AOR	p	95% CI
**Age** (years)		0.41	0.061	0.16–1.04	0.39	0.025	0.17–0.89
**Education level**	Less than 4 years of nursing education (Ref.)	1			1		
	More than 4 years of nursing education	1.39	0.595	0.41–4.75	0.86	0.768	0.31–2.39
**Workplace^a^**	Health or Prevention (Ref.)	1			1		
	Medical	1.04	0.958	0.28–3.87	1.62	0.421	0.50–5.21
**Changing self-efficacy after 3 months^b^**	Negative change or no change (Ref.)	1			1		
	Positive change	2.39	0.049	1.00–5.69	2.54	0.02	1.16–5.60
**Implementation status of ‘Ask’ before the program**	Rarely, never (Ref.)	1			1		
	Always, usually, sometime	0.90	0.859	0.28–2.85	1.17	0.801	0.36–3.82
**Implementation status of ‘Advise’ before the program**	Rarely, never (Ref.)	1			1		
	Always, usually, sometime	3.68	0.008	1.40–9.65	2.77	0.031	1.10–7.01
**Implementation status of ‘Assess’ before the program**	Rarely, never (Ref.)	-	-	-	1		
	Always, usually, sometime	-	-	-	1.93	0.109	0.86–4.31
**Changing self-efficacy post-program** (not adjusted)	Negative change or no change (Ref.)	1			1		
	Positive change	1.18	0.760	0.42–3.31	0.89	0.809	0.33–2.36

a Health or Prevention: public health center or health check-up center. Medical: hospital or clinic.

b Changing self-efficacy for smoking cessation counseling 3 months after the program.

AOR: adjusted odds ratio; adjustment for age, education level (less than vs more than 4 years of nursing education), workplace (health or prevention workplace such as public health center or health check-up center vs medical workplace such as hospital or clinic), changing self-efficacy for smoking cessation counseling before and after 3 months the program (negative vs positive-efficacy group), and implementation status of ‘Ask’, ‘Advise’, ‘Assess’ before the program (negative vs positive).

## DISCUSSION

This study found that a one-day smoking cessation educational program contributed to increasing the frequency of Japanese nurses providing smoking cessation counseling among those who had previously infrequently advised patients according to the 5As of the clinical practice guidelines for treating tobacco use and dependence. Nurses who had infrequently assessed the readiness of patients to quit smoking before they participated in the educational program had significantly increased this activity 3 months thereafter. Increase of self-efficacy for smoking cessation counseling and experience of having previously delivered the ‘Advise’ item to encourage patients to quit smoking were positively associated with increased frequency of employing the ‘Assess’ and ‘Assist’ items thereafter.

The effectiveness of this program was evaluated by assessing nurses’ behavioral changes using the 5As method. Previously, only a few studies had evaluated the efficacy of smoking cessation educational programs for nurses by comparing their behavior before and after participating in such a program^14,16,20^. Different from our approach, Barta et al.^15^ reported on a 2-hour brief intervention training program based on the 5As, but only with 20 nurses, to assess changing self-efficacy and practices 6 weeks after the program. The results suggested a positive effect of the program on nurses’ smoking cessation counseling evaluated through the 5As, except for the ‘Ask’ item^15^. The authors suggested that this latter finding may be attributed to the nurses already having asked about the patients’ smoking status during their routine admission assessment. As in that study^15^, most nurses who participated in our program were already consistently involved in screening patients’ tobacco use prior to taking part in the program.

In another study, Hughes et al.^14^ first delivered a 20 minute lecture on the 5As and provided participants with a 5As Pocket Guide. The 36 nurses in that study were not found to have improved their knowledge and delivery of 5As-based counseling 4 weeks after the program. The authors mentioned that little practice and repetition after the program may have prevented reinforcement of knowledge retention and skill acquisition^14^. The content of the educational programs in most previous studies was focused on how to use the 5As in a short time.

Finally, Gonzalez et al.^20^ used a 2-hour educational program for smoking cessation counseling for 42 nurses working in an Emergency Department. This educational program consisted of the patient flagging a chart of tobacco use, smoking cessation counseling for patients, providing telephone quitline numbers, and referring patients to community-based services and for pharmacological assistance. Results suggested that nurses’ smoking cessation delivery of ‘Assess’, ‘Assist’, and ‘Arrange’ was changed positively 2 weeks after the program^20^. The authors noted that the participants already had relatively high levels of delivering advice to quit at baseline, and this did not change significantly as a result of the program. Further steps (Assess, Assist, Arrange) were not routinely implemented to help patients quit smoking^20^. This program consisted of practical smoking cessation education to help nurses who worked in an Emergency Department. However, the follow-up period was only 2 weeks. This might be too short a time to accurately assess the effectiveness of the educational program. In addition, most studies that evaluated the efficacy of nurses’ smoking cessation educational programs reported on less than 100 subjects. In contrast, our study comprised 289 participants from 13 prefectures across Japan surveyed before and 3 months after the educational program. Our program was also longer and its content also included more practical skill training over 6 hours.

Those participants already delivering ‘Advise’ for patients to quit smoking showed significant positive behavioral change for both the ‘Assess’ and ‘Assist’ items 3 months thereafter. The results of an RCT using an e-learning program for nurses’ smoking cessation education suggested that nurses with more than the average number of years of counseling experience delivered better smoking cessation advice in practice^21^. We considered that participants who already exhibited a readiness for routinely delivering ‘Ask’ were more likely to be encouraged to go to the next steps of ‘Assess’ and ‘Assist’ after the program.

Increased self-efficacy for the delivery of smoking cessation counseling 3 months after the program was significantly associated with positive behavioral changes. However, increased self-efficacy after participating in the program was not related to the nurses’ practice 3 months after the program. It seems that increasing self-efficacy facilitated by the program was not related to continuing the changes towards ‘Assess’ and ‘Assist’. This indicates that it is important to continue to increase self-efficacy in the long-term after the program. Bandura^22^ developed the self-efficacy concept and demonstrated that expectations of personal efficacy are derived from performance accomplishments. Self-efficacy and practice are expected to interact and mutually increase. For this reason, it was considered that the smoking cessation program developed here should include practical content, necessary to improve self-efficacy while practicing smoking cessation counseling.

Regarding the implementation rate of the 5As by nurses, in a study of 152 Czechoslovak nurses, Sarna et al.11 reported that the prevalence of using 5As in real-world practice was 63.0% for ‘Ask’, 45.8% ‘Advise’, 38.9% ‘Assess’, 26.0% ‘Assist’ and only 11.8% for ‘Arrange’. Another study on 507 Eastern European nurses found that the prevalence of practicing the 5As was ‘Ask’ 70.4%, ‘Advise’ 65.7%, ‘Assess’ 58.6%, ‘Assist’ 36.3%, and ‘Arrange’ 20.4%^23^. In contrast, in Japan, a survey of 554 public health nurses by Li et al.^24^ indicated that the frequency of ‘Ask’ was as high as 83.9%, ‘Advice’ 58.0%, ‘Assess’ 24.3%, ‘Assist’ 32.5%, and ‘Arrange’ very high at 43.9%. Taken together, these results indicate that nurses’ implementation rate of the item ‘Ask’ is generally quite high worldwide, but the other four are much lower. From these results, we believe that programs aimed at increasing the delivery rate of ‘Assess’, ‘Assist’ and ‘Arrange’, such as our program, are important for increasing the implementation rate of smoking cessation counseling by nurses.

### Limitations

A potential limitation of our study is the representativeness of the sample of participants tested. The response rate 3 months after the program was only 46.0%, potentially biasing the result in favor of those for whom the intervention was most successful in changing behavior for reasons such as exposure to greater opportunities to apply the 5As counseling technique. Also, we investigated only Japanese nurses’ practices. A previous study that investigated the smoking cessation practices and beliefs of oncology nurses in six countries suggested that Japanese nurses’ practice of smoking cessation counseling was more conservative compared to other countries^25^. Therefore, these findings may not apply to nurses in other countries. Finally, our study did not analyze perceptional changes in smoking cessation. Participants who changed their behavior may have changed their perceptions of smoking cessation counseling through the program. Further studies are needed to determine detailed nurses’ perceptions.

## CONCLUSIONS

A one-day smoking cessation educational program was effective at increasing the frequency of Japanese nurses providing smoking cessation counseling. Greater readiness to deliver smoking cessation counseling before participating in the educational program and to continue at a higher level of self-efficacy while practicing thereafter is important to encourage a behavioral change for delivering smoking cessation counseling.

## Data Availability

The data supporting this research cannot be made available for privacy or other reasons.
